# Malignant and Noninvasive Skin Tumours in Renal Transplant Recipients

**DOI:** 10.1155/2014/409058

**Published:** 2014-09-14

**Authors:** Christopher D. Roche, Joelle S. Dobson, Sion K. Williams, Mara Quante, Joyce Popoola, Jade W. M. Chow

**Affiliations:** ^1^St George's, University of London, Cranmer Terrace, London SW17 0RE, UK; ^2^The National Hospital for Neurology, 23 Queen Square, London WC1N 3BG, UK; ^3^Department of Histopathology, Royal Sussex County Hospital, Eastern Road, Brighton BN2 5BE, UK; ^4^Department of Renal Medicine and Transplantation, St George's Healthcare NHS Trust, Blackshaw Road, London SW17 0QT, UK; ^5^International Medical University, Jalan Jalil Perkasa 19, 57000 Kuala Lumpur, Malaysia

## Abstract

*Background.* Transplant recipients require immunosuppression to prevent graft rejection. This conveys an increased risk of malignancy, particularly skin tumours. There is a need for up-to-date data for the South of England. *Method.* Pathology records were reviewed for 709 kidney transplant recipients on immunosuppression at our hospital from 1995 to 2008. Skin tumours were recorded/analysed. *Results.* Mean age at transplant was 46 years. Mean length of follow-up was 7.2 years and total follow-up was 4926 person-years. 53 (7.5%) patients (39/458 (8.5%) males and 14/251 (5.6%) females) developed ≥1 skin malignancy. Cumulative incidences of 4.0%, 7.5%, and 12.2% were observed for those with <5, <10, and ≥10 years follow-up, respectively. The rate was 45 tumours per 1000 person-years at risk. Additionally, 21 patients (3.0%) only had noninvasive tumours. 221 malignant skin tumours were found: 50.2% were SCCs, 47.1% BCCs, and 2.7% malignant melanomas. Mean years to first tumour were 5.8. Mean number of tumours per patient was 4, with mean interval of 12 months. *Conclusions.* Despite changes in transplantation practice during the time since the last data were published in this region, these findings are similar to previous studies. This adds to the evidence allowing clinicians to inform patients in this region of their risk.

## 1. Introduction

Organ transplant recipients take immunosuppressive drugs for life to prevent graft rejection. This renders them at increased risk of many types of malignancy and they have been found to be between four [1] and fourteen [2] times more likely to develop cancer than age-matched controls. They are particularly at risk of developing nonmelanoma skin cancer (NMSC). One RCT reported that, over 20 years, 35% of their transplant patients developed NMSC alone, when for all other types of cancer the combined cumulative incidence was 19% [3]. Another study found that skin cancers accounted for 88% of cancers in the immunosuppressed patients, with the next most frequent single malignancy being lymphoma, at 1.4% [2]. Renal transplant recipients have been the most extensively studied but the risk of malignancy applies to all patients receiving long-term immunosuppression. The cumulative dose of immunosuppression over time has been found to be the primary factor driving the tendency towards malignancy [2–10]. The other strong risk factor is UV light exposure in fair-skinned transplant patients [3, 11–15]. After 20 years of follow-up, the prevalence of NMSC for these patients has been reported to be as high as 41% in the Netherlands [14], 61% in the UK [11], and 82% in Australia [15].

In the South of England, there is a relatively low UV light burden but many patients with light, high-risk skin types. Previous regional studies have reported cumulative incidences of 5% in Oxford (1989) [16], 19% in Oxford (2004) [11], and 22% in London (1994) [17]. In the past, there was a lack of patient knowledge [18, 19], but now that more is known about the risk of sun exposure, it might be expected that patients would be better informed about the need for sun protection and clinicians would be more efficient in surveillance and detection. It is not known whether the rates have changed in this region as, in UK, there have been fewer than 10 published studies since 1987, with the most recent one in 2007 [20] and the most recent London study in 1994 [17].

With the significant advance in transplantation medicine, it is important to have up-to-date local data to inform practice. The aims of this study are to determine the risk of developing skin cancer, including noninvasive tumours, for renal transplant recipients. In addition, it will assess whether this has changed since historical published data and provide current data for the region to inform patients and clinicians.

## 2. Materials and Methods

A list of all patients receiving a renal transplant at St George's Hospital (SGH) between 1995 and 2008 was obtained and demographic data were recorded onto a spreadsheet. Where the transplant from this list was not the patient's first one, the date of their first transplant was obtained and used as the start date of their immunosuppression, with the earliest case being in 1986. The date of the most recent hospital attendance or blood test was used to mark the last date of follow-up, unless the patient had died, in which case the date of death was used.

The electronic pathology reports for each patient were reviewed at each of the three hospitals where the patients were followed up. All malignant (BCC, SCC, malignant melanoma (MM)) skin tumours developed after the dates of transplant were recorded. For completeness, noninvasive tumours (Bowen's disease (SCC in situ) and actinic keratosis) were also recorded. Keratoacanthoma behaves like SCC in immunosuppressed patients and for the purposes of this study, these were classified as well-differentiated SCC. The date of the pathology report, site of the tumour, subtype of BCC, grade of SCC, and Breslow thickness for MM were recorded.

The scope of this study was limited to skin tumours, and nonskin malignancies were not recorded or analysed. The three cohorts followed up at three sites were analysed separately and appeared similar; therefore, the combined data are analysed and those results are shown. Statistical testing was conducted with Quickcalcs (Graphpad) and XLSTAT (Addinsoft) and Fisher's exact test was used to test significance. This project was reviewed and approved by (SGH), Audit Department, and assigned a project number (DB314).

## 3. Results

Demographic data are shown in Table 1. There were 769 patients who received a kidney transplant at SGH, London, between 1995 and 2008. 20 (2.6%) transferred out of the area, 15 (2.0%) were removed from the sample for having less than 3 months follow-up time, and 25 (3.3%) were lost to follow-up. The remaining 709 patients were all followed up in South East England with 346 at SGH, 192 at St Helier Hospital, London, and 171 at the Royal Sussex County Hospital, Brighton. On average, the medical records for these patients covered 84.3% of the maximum achievable follow-up time.

**Figure 1:**
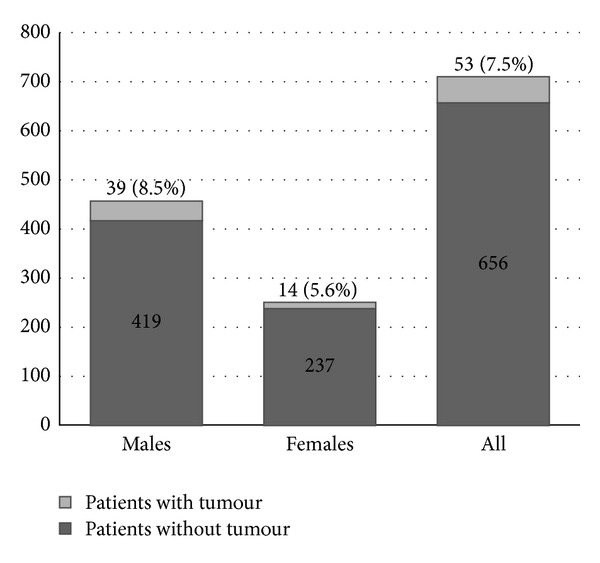
The absolute numbers and percentages of male and female kidney transplant recipients in our study population who received a renal transplant between 1995 and 2008 and had developed a malignant skin tumour at the time of data collection.

53 of 709 patients (7.5%) had at least one malignant skin tumour (Table 1). The rate of skin cancer was 45 per 1000 person-years at risk. 39 of 458 males (8.5%) and 14 of 251 females (5.6%) had at least one malignant tumour (OR 1.6, *P* = 0.18) (Figure 1). 49 (6.9%) had an NMSC. 36 (5.1%) had at least one BCC and 21 (3.0%) had an SCC, with 8 patients (11.3%) having both tumour types. 6 patients (0.8%) had an MM, with 2 (0.3%) having both MM and NMSC.

Additionally, 21 (3.0%) only developed noninvasive tumours (Bowen's disease (BD) and actinic keratosis (AK)) which may progress to invasive SCC. For those noninvasive tumours, there were 31 patients with 88 tumours. Of these, 21 never developed an SCC and these patients had a total of 23 of the noninvasive skin tumours described above.

**Figure 2:**
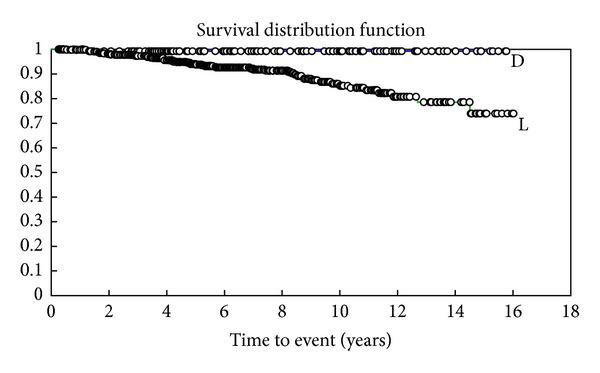
Kaplan Meier survival curve shows two groups—patients with Fitzpatrick skin types I–III (light-skinned—L) with worse times to first malignant tumour and patients with Fitzpatrick types IV–VI (dark skinned—D) where only one malignant tumour occurred.

Only one (1.9%) of the patients who developed a skin tumour following transplantation was nonwhite (Fitzpatrick skin types IV–VI) (Figure 2), compared with the ethnic mix of the local transplant population being 64% white, 14% African or Caribbean, 13% South Asian, 6% East Asian, and 3% from other ethnic groups. The rate of skin cancer for white transplant patients was 57 per 1000 person-years at risk.

Of the 247 patients with 3-month to 5-year follow-up, 10 (4.0%) developed a total of 22 malignant skin tumours (12 BCCs, 10 SCCs); of the 281 with 5–10-year follow-up, 21 (7.5%) developed a total of 49 tumours (36 BCCs, 9 SCCs, 4 MMs); of the 181 patients with over 10-year follow-up 22 (12.2%) developed 150 tumours (56 BCCs, 92 SCCs, 2 MMs). This last group included a patient who had his original transplant in 1986. After 25 years of immunosuppression, he developed 55 SCCs, 21 noninvasive tumours (BD or AK), and 2 BCCs between 1996 and 2011. Another notable patient had 8 pretransplant BCCs over 6 years and developed 22 posttransplant BCCs and none of the other tumour types over his 9 years of posttransplant follow-up.

**Figure 3:**
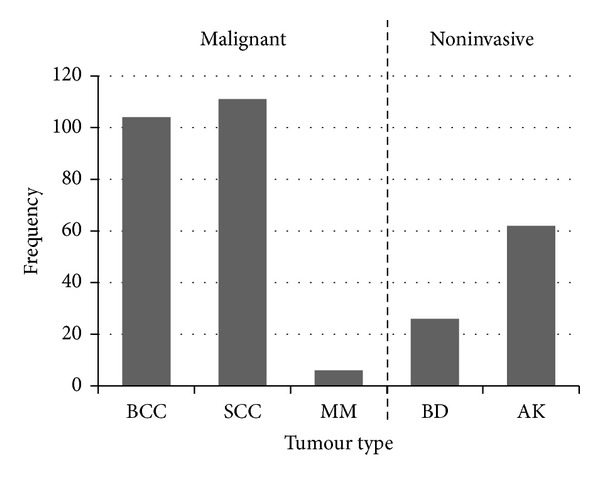
Frequency of skin tumour types among renal transplant recipients in the study population.

Of the 221 malignant tumours that were found, 104 (47.1%) were BCC, 111 (50.2%) were SCC, and 6 (2.7%) were MM. In addition, there were 88 noninvasive tumours, of which 26 were BD and 62 were AK (Figure 3).

**Figure 4:**
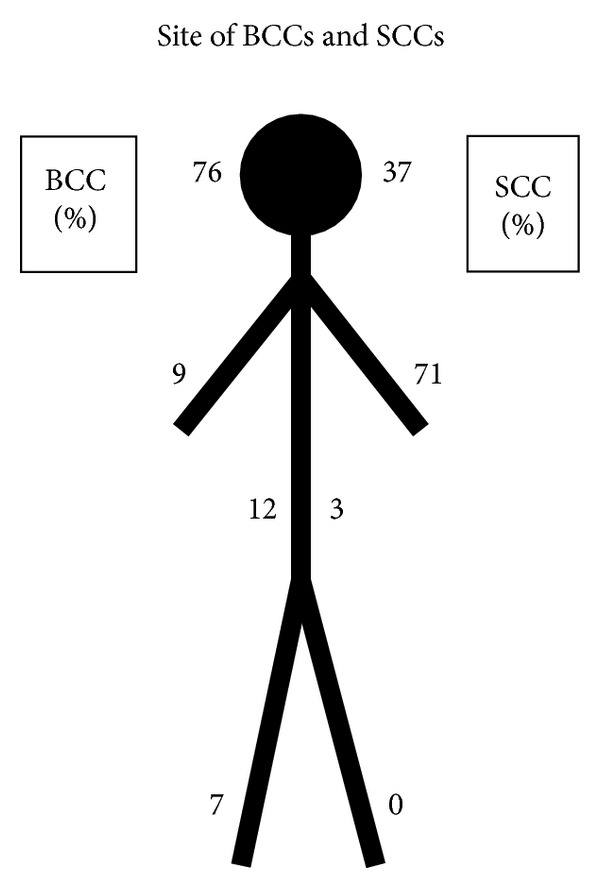
Anatomical distribution of NMSC tumours shows predominance in sun-exposed sites.

Of the BCCs, 76 were on the head and neck, 12 on the trunk, 9 on the upper limb, and 7 on the lower limb. Of the SCCs, 37 were on the head and neck, 3 on the trunk, 71 on the upper limb, and none on the lower limb (Figure 4). Of the noninvasive tumours, 33 were on the head and neck, 8 on the trunk, 38 on the upper limb, and 8 on the lower limb and in one case the location was not documented. Of the MMs, 1 was on the head and neck, 4 on the trunk, none on the upper limb, and 1 on the lower limb.

For BCC subtype, 63 (61%) were nodular; 16 (15%) were superficial type; 14 (13%) were infiltrative; 1 (1%) was basosquamous; and for 10 (10%) the subtype was not documented.

For SCC grade, 53 (48%) were well differentiated; 29 (26%) were moderately differentiated; 1 (1%) was poorly differentiated and for 28 SCCs (25%) the grade was not documented.

For the thickness of the 6 MMs, 3 were <1 mm; 1 was 1-2 mm, and 2 were 2–4 mm.

## 4. Discussion

Cumulative incidences of skin tumours following renal transplantation reported in the literature have varied according to length of follow-up of the sample and the inclusion criteria for tumours in the analysis. The 7.5% cumulative incidence of NMSC (10.5% if noninvasive tumours are added) observed in this study is comparable to those observed in other studies of similar design and follow-up period [11, 14, 16, 17].

Whilst only 4% of patients at <5 years follow-up developed a tumour, our department begins annual screening immediately after transplant as recommended by* The American Society of Transplantation* [21]. Our observed cumulative incidences of 7.5% and 12.2% for those with >5- and >10-year follow-up, respectively, contrast with 23% (>5 years) and 44% (>9 years) in sunlight-rich Australia [22]. This illustrates the importance of sun avoidance and annual screening is an opportunity to identify high-risk patients and impart education.

The BCC : SCC ratio was equalized at 1 : 1, compared to 7 : 1 in the normal population [23, 24], showing the particular risk of SCC for transplant patients. This is thought to be related to HPV which is known to be an important aetiological factor in SCC but not for BCC or MM (whereas sun exposure is an aetiological factor for all three). The precise mechanism is not clear and is the subject of some debate. There is some evidence that HPV infection, which is often transient, damages cellular DNA in a “hit and run” fashion, not by genomic integration as it does for cervical cancer [25, 26]. However, HPV genes such as E6 and E7 have been found to persist in the DNA of host SCCs, suggesting that at least some element of the pathogenesis is not entirely transient [27]. Ultimately HPV is thought to interfere with tumour suppressor genes such as p53 which would normally initiate apoptosis or repair genetic damage caused, for instance, by UV light [8, 17]. Importantly for immunosuppressed patients, they may be more susceptible to acquiring HPV infection in the first place, with 90% of transplant patients infected at a given time [28]. Furthermore, HPV types which are not known to be oncogenic (e.g., HPV 1/2) may become so in transplant patients and oncogenic strains (e.g., HPV 16/18) which are normally confined to the mucosa may manifest in the skin of transplant patients [29].

This study did not explore the role of different immunosuppressive drugs as these have been compared extensively elsewhere with no advantage between the commonly used regimes [3, 30]. The majority of our renal transplant recipients were on a calcineurin inhibitor (tacrolimus or cyclosporine) with either prednisolone or an antiproliferative agent (mycophenolate mofetil or azathioprine). There is emerging evidence to suggest that the newer m-Tor inhibitors (sirolimus/everolimus) may confer an advantage in reducing de novo malignancies in comparison to calcineurin inhibitors [4–6], though this finding applies to organ transplant recipients with a low and early SCC burden only. Longer-term evidence for the newer agents will emerge in the future, but current evidence suggests that the cumulative immunosuppressive load remains the most important risk factor [3].

Many of the limitations of this study are those inherent to the retrospective study design. In particular, it is not certain that all tumours were accounted for, as there may have been tumours which did not present to our service and therefore would not have been on the hospital records (e.g., if a patient presented to their GP and a sample was not taken). However, this scenario can be expected to be rare because of the education given to our GPs around the increased risks associated with skin malignancy in patients on immunosuppression.

Because this is a case series and the cohort is not compared to matched controls, the obtained figures are open to fluctuations depending on the nature of this cohort and baseline population rates are not accounted for. Confounding factors within the cohort such as the proportion with fair skin, age at transplant, length of follow-up, and underlying genetic susceptibilities may have influenced the results. The only randomised control trial (RCT) on this subject was recently published in Australia and found an overall NMSC cumulative incidence of 35% at 20-year median follow-up [3]. This is lower than previous retrospective studies which have been reported, even in low-sunlight areas such as Oxford where 61% was reported [10]. The cumulative incidence observed in our study approximates to the RCT's predicted incidence for low-risk patients, which was 1.9% at 5 years, 4.2% at 10 years, and 9.3% thereafter [3]. This would be expected in a region of lower sun exposure such as UK and supports the validity of our results.

Our findings being slightly lower than some previous UK studies may show the impact of patient education, as the 20-year follow-up patients in previous retrospective cohorts were transplanted before the era of increased awareness of the risk of sun exposure (e.g., the highly successful Australian “Slip! Slap! Slop!” public health campaign started in 1981) which is not the case in the RCT cohort recruited between 1983 and 1986. It also shows the importance of individual patient susceptibility, as 77% of patients in the RCT had olive or darker, less susceptible skin types (indeed the authors predict a 99% rate for fair-skinned high-risk patients). Transplant patients should be educated to understand their particular danger from sun exposure and they should avoid direct sunlight and use sun protection factor 50. Skin color is important, as highlighted by the low risk in patients in our cohort with skin types IV–VI (all but one of the malignant tumours in our cohort were seen in patients with skin types I–III). The variability in risk between different patients should be formally assessed by clinicians, with high-risk patients identified for increased surveillance.

## 5. Conclusion

This study provides recent data for London and South East England. Despite changes in transplantation practice during the time since the last data were published in this region, these findings are similar to those of previous studies. This study adds to the evidence allowing clinicians in temperate regions to inform patients of their risk of developing skin tumours after renal transplantation.

## Figures and Tables

**Table 1:** Patient demographic data and results at a glance. The results show the mean length of follow-up from time of transplant to time of the most recent electronic entry to the patient's record, the interval between tumours in those that developed at least two, and the mean number of tumours developed.

Demographics and results at a glance	
Patients, *n*	709
Males, *n* (%)	458 (65)
Females, *n* (%)	251 (35)
Mean length follow-up, yrs (SD)	7.2 (3.9)
Total follow-up in person-years	4926
Mean age at transplant, yrs (SD)	46 (13.0)
Patients with skin types I–III (white), *n* (%)	567 (80)
Patients with malignant skin tumour, *n* (%)	53 (7.5)
Mean time to 1st tumour, yrs (SD)	5.8 (3.3)
Mean tumours per patient (SD)	4 (8.5)
Mean tumour interval, yrs (SD)	1 (1.4)

## Abbreviations

AK: Actinic keratosis
BCC: Basal cell carcinoma
BD: Bowen's disease
HPV: Human papilloma virus
MM: Malignant melanoma
NMSC: Nonmelanoma skin cancer
RCT: Randomised control trial
SCC: Squamous cell carcinoma
SGH: St George's Hospital.
